# Factors influencing management of dry cell battery waste: a case of Greater Accra Region in Ghana

**DOI:** 10.1007/s10661-024-13297-4

**Published:** 2024-11-08

**Authors:** Justice Kofi Debrah, Godfred Kwesi Teye, Maria Alzira Pimenta Dinis

**Affiliations:** 1 Faculty of Science and Technology, University Fernando Pessoa (UFP), Praça 9 de Abril 349, 4249-004 Porto, Portugal; 2 Key Laboratory of Integrated Regulation and Resource Development On Shallow Lakes of Ministry of Education, College of Environment, Hohai University, No. 1 Xikang Road, Nanjing, 210098 China; 3 Fernando Pessoa Research, Innovation and Development Institute (FP-I3ID), University Fernando Pessoa (UFP), Praça 9 de Abril 349, 4249-004 Porto, Portugal; 4 Marine and Environmental Sciences Centre (MARE), University of Coimbra, Edifício do Patronato, Rua da Matemática, 49, 3004-517 Coimbra, Portugal

**Keywords:** Dry cell battery (DCB) waste, Waste management (WM), Sustainability, Greater Accra Region, Ghana

## Abstract

Indiscriminate disposal of dry cell battery (DCB) waste contributes to environmental and public health issues in developing countries such as Ghana, due to the toxic nature of this specific waste. Accordingly, a study was conducted in Accra, Ghana, to determine the socio-economic and demographic factors influencing handling DCB waste, aiming a sustainable environment. Using a random sampling technique, a descriptive cross-sectional survey was conducted, encompassing 367 respondents from the Accra-Tema Metropolitan areas and Tema West Municipal Assembly in Greater Accra, Ghana. Using descriptive and multivariate statistical methods, the survey data were analysed with the Statistical Package for Social Sciences (SPSS) version 27. The results of this study show that female gender and residential area are likely to positively influence the use of DCB at home. Education significantly affects the use of DCB and its proper disposal. The results also suggest that 78% of the respondents disposed of DCB waste in waste bins. The mean monthly income of the respondents stands at USD 270, which is average and likely partially to positively influence the disposal of the DCB. The data collected revealed that female gender, age group, family size, and education level influence the indiscriminate disposal of DCB waste and DCB waste recycling. The results highlight that educated females above the age of 55, with a monthly income, are likely to properly segregate DCB waste. This study contributes to the knowledge gap in relation to dry cell battery waste management (DCBWM) in developing countries, aiming to advance global sustainability. This study is expected to contribute to educate and create awareness in managing DCB waste to reduce its indiscriminate disposal which leads to environmental pollution and negatively affects human health and environmental sustainability in Ghana.

## Introduction

Global waste production, in particular resulting from the use of dry cell battery (DCB), such as alkaline, zinc-carbon, and lithium-ion, has steadily increased, resulting in a rise in the volume of electronic waste (Borchers et al., 2021; Toro et al., 2023). Dry cell battery waste management (DCBWM) challenge is due to the lack of comprehensive legislation, absence of infrastructure development, and lack of public awareness efforts to improve collection and recycling in most nations (Ferronato & Torretta, 2019; Gianvincenzi et al., 2024; Kang et al., 2023; Zanoletti et al., 2024). Some countries have implemented rules and regulations in regard to collecting and recycling, resulting in less than 50% of DCB waste being recycled worldwide (Dobó et al., 2023). However, due to the lack of a global framework and infrastructural development plan in most countries, DCB waste disposal is not properly carried out.

In sub-Saharan Africa (SSA), DCBWM will continue to be a significant environmental issue. DCB is used to comply to basic needs of human life, such as torchlights, radios and remote controls (Mamady, 2016). Studies by Malav et al. (2020) and Mohan and Joseph (2021) have reported that industrialization and population growth are the causes of the poor management of DCB waste in most developing countries. Also, a review study by Debrah et al. (2022a) on factors limiting the progress of circular economy in SSA and another study from Venkiteela (2020) about the status and challenges of solid waste management (WM) in Tirupati City, Southern India, have revealed that increasing population growth gives rise to waste generation including DCB waste. A research conducted by Adedara et al. (2023) revealed that already in 2016, SSA countries generated 191.8 million tons of waste, corresponding to a population of 1.04 billion (The World Bank, IBRD-IDA, Data, 2023), which has increased to 198.4 million tons and a population of 1.31 billion in 2019 (Ayeleru et al., 2020; Debrah et al., 2022a). According to Scarlat et al. (2015), SSA waste was expected to increase to 244 million tons by 2025 and a 1.5 billion population size (United Nations - World Population Prospects, 2022). However, even with this exponential rate of waste generation in SSA countries, only 44% is collected and disposed of in landfills (Hoornweg & Bhada-Tata, 2012), with the remaining improperly disposed due to lack of funding, tools and logistics challenges (Debrah et al., 2022a, b, c, d, e, 2023). Improper management of DCB waste threatens the ecological system and public health (Abubakar et al., 2022; Debrah et al., 2021a, b, c, 2022a, b, c, d, e; Masud et al., 2019), affecting Sustainable Development Goals (SDGs) progress (Debrah et al., 2022a; Kumar et al., 2021; Taušová et al., 2019), such as good health (SDG 3), environmental sanitation (SDG 6), and sustainable cities and communities (SDG 11) (United Nations, 2020). The poor WM process in SSA and other developing countries results from lack of adequate infrastructure for recycling (Debrah et al., 2022a, b, c, d, e; Filho et al., 2022a; Godfrey, 2019), absence of modern technology (Leal Filho et al., 2023a), inadequate knowledgeable human resources (Debrah et al., 2021b, 2022a, b, c, d, e; Khan et al., 2019; Rautela et al., 2021), and a lack of policy and enforcement of law and regulations (Muheirwe et al., 2022). Furthermore, more than 65% of waste generated, including plastic, is not segregated within SSA countries (Ayeleru et al., 2020; Kaza et al., 2018). This unsegregated waste contains hazardous waste like DCB waste and is disposed of at either in unsanitary landfills, burnt in any space within the community, or buried (Rasmeni & Madyira, 2019; Salam et al., 2021), negatively impacting on climate change (SDG 13), life below water (SDG 14) and life on land (SDG 15) (Leal Filho et al., 2023b), among others.

DCB is considered hazardous if mismanaged due to the chemical component that is converted to electrical energy for power (Rupani et al., 2019). In Africa, most citizens use the DCB as source of energy since an average of 550 million people lack access to electricity coupled with regular interruption of energy supply (International Energy Agency, 2022; World Bank/IEA, 2015) at home. Poor handling and management of these DCB waste can result in lead (Pb) poisoning of soil, affecting plants (Kohli et al., 2020; Zulfiqar et al., 2019) and high blood Pb levels in humans (Etiang et al., 2018; Ondayo et al., 2016; Arias et al., 2018). Some chemical elements that make DCB hazardous include cadmium (Cd), zinc (Zn), manganese (Mn), nickel (Ni), silver (Ag), mercury (Hg), and other chemicals such as ammonium chloride (CH_4_Cl) and dichloroisocyanuric acid (C_3_HCl_2_N_3_O_3_) (Dehghani-Sanij et al., 2019; Meshram et al., 2020) with lithium (Li) posing fire threat (Ouyang et al., 2017; Peng et al., 2020).

Although different kinds of batteries are available, this study focuses on DCB, predominantly used in households and found in devices such as telephones, radios, remote controls, toys, flashlights, games, watches, calculators, and cameras, as in many other devices (Environmental Affairs and Republic of South Africa, 2018). In Ghana, managing DCB waste has become an issue since it is not segregated from other domestic waste and is able to cause skin irritation and affect growth, compromising the quality of life (SDG 3) (Mrozik et al., 2021), among all other issues and SDGSs already mentioned. DCB waste is disposed of in landfills, dumped in any available space, or buried, adversely affecting living organisms and the environment. Cd present in the DCB reduces organic waste degradation and destroys soil microorganisms (Alengebawy et al., 2021; Wang et al., 2020). Also, excess Cd in water bioaccumulates and changes fish’s reproductive and physiological behaviours (Fakhri et al., 2021; Per et al., 2015; Renieri et al., 2014), making fish unhealthy for consumption and negatively affecting production patterns (SDG 12).

Studies by Purcell and Magette (2010) and by Mamun and Hossain, (2021) reported that socio-economic status and demographic characterisation could determine locality or residential community of participants, influencing the type and quantity of waste generated and its management. Appropriate measures for collection and disposal must be available. Knowledge, attitude, and awareness in handling waste could lead to effective and successful practices toward sustainable DCBWM (Debrah et al., 2021b; Tolera et al., 2022). Accordingly, this study is expected to contribute to identify the factors associated with DCB mismanagement in Ghana. It further aims to assist decision-making by WM experts to implement sustainable DCBWM in developing countries and, Ghana, in particular. This research will significantly contribute to raise awareness of the proper management of DCB waste to enhance a healthier environment.

## Socio-demographic and socio-economic characterisation of the sample

WM at the household level is greatly affected by the demographic characterisations and socio-economic conditions in Africa, e.g., education, age, gender, income, residential area and marital status (Suárez-Perales et al., 2021). According to Mamady (2016), in Guinea, residential communities can be classified as *planned* and *unplanned*. A planned community has a good road network, stores, water, and other amenities, making living enjoyable, while unplanned communities lack road networks, absence of stores, and other social amenities (Licavoli, 2021; Luo & Yang, 2021). In light of it, demographic characterisation, especially education, is a critical factor that might directly influence many areas of human life (Haleem et al., 2022; Zareie and Navimipour, 2016), such as DCBWM. Socio-demography factors such as educational level, age, and income significantly influence WM practices at the point of generation, especially at the household level (Jribi et al., 2020), assisting in protecting the environment (Debrah et al., 2021b; Estrada-Vidal et al., 2020). Education creates awareness and enhances better understanding of DCBWM, resulting in a healthier environment through proper DCBWM practices (Babaei et al., 2015; Campos & Fernandes, 2020; Hasan, 2004) for better social engagement of WM (Carlotto et al., 2022; Debrah et al., 2022d). It also provides knowledge of DCBWM techniques, reducing the volume of waste that ends up in landfills, for the possible achievement of SDGs for good health and well-being (SGD 3), clean water and sanitation (SDG 6), and sustainable cities and communities (SDG 11). Also, for sustainable consumption and production pattern (SDG 12), climate action (SDG 13), life below water (SDG 14) and for life on land (SDG 15). According to Hadjichambis et al. (2020), individuals with higher levels of education may have better critical thinking skills and problem-solving abilities that identify effective DCBWM solutions. Also, education serves a vital role in promoting sustainable WM practices at the household level (Boca & Saraçli, 2019; Gilal et al., 2019), particularly in the context of developing countries. Socio-demographic factors such as continuing education enhance knowledge, skills, and awareness of proper WM, assisting in reducing DCB waste generation and in protecting the environment.

The socio-economic settings largely impact waste generation in most societies. According to Cheng et al. (2020), affluent communities have a high waste generation due to high purchasing power and pattern of consumption, resulting into an increase of waste collection activities. The volume and composition of waste including DCB are influenced by economic level of inhabitants, requiring tools and logistics and financial commitment for proper WM (Haleem et al., 2022). Consequently, irregular collections of waste including DCB waste could lead to accumulation of toxic chemicals (Ali et al., 2019), posing significant threats to the environment and human health.

## Research methods

### Study area

**Fig. 1:**
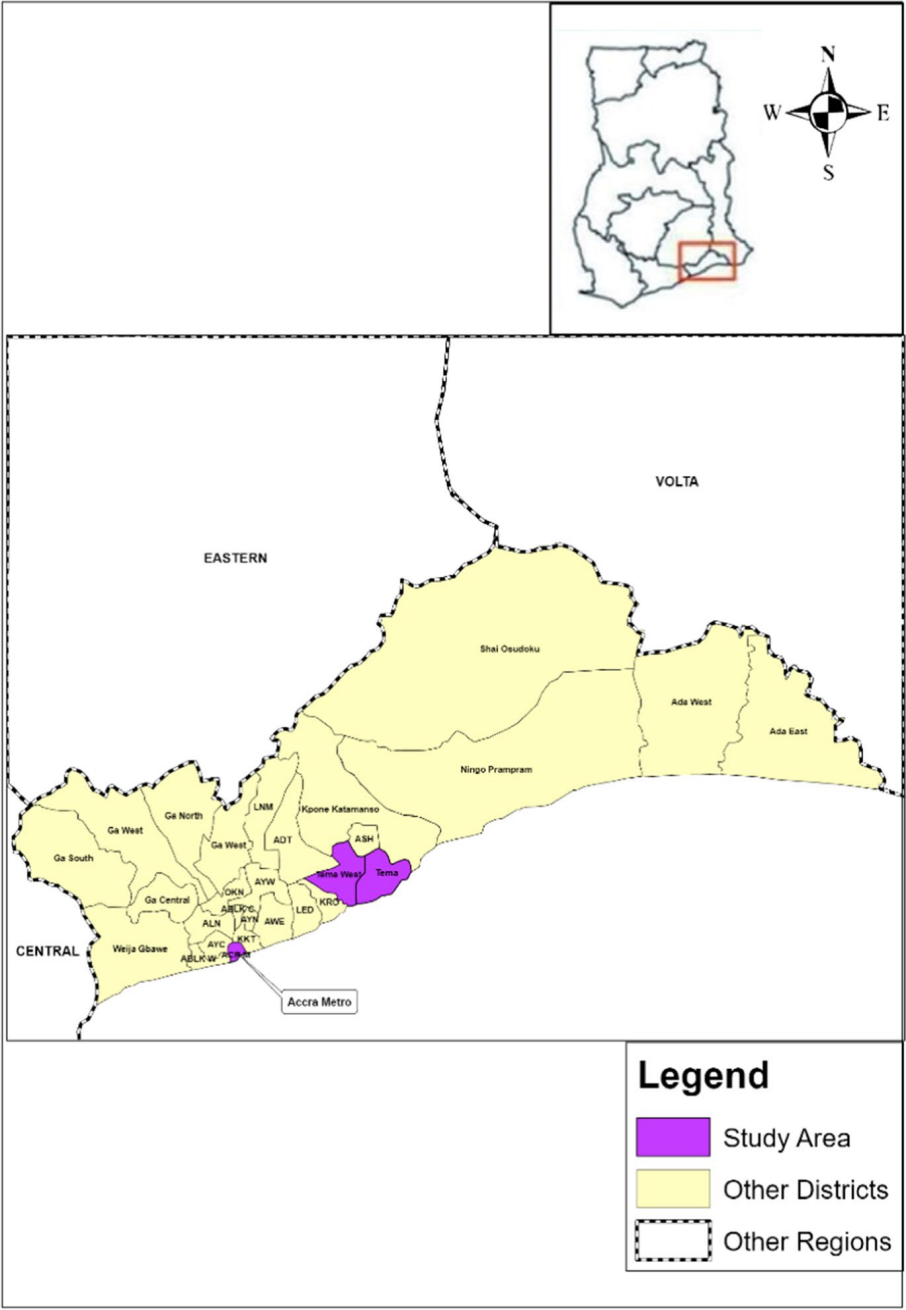
Shaded Greater Accra Regional map with the study area

This study was conducted in parts of the Greater Accra Region, which includes Accra-Tema Metropolitan and Tema West Municipal Assembly, which covers a large part of the central and coastal areas of the region, as shown in Fig. 1. The research questionnaire was administered in the selected area, with a population size of 658,272 out of 5,455,692 (Greater Accra Region), representing 12% of the total population of the Greater Accra Region (Ghana Statistical Service (GSS), 2021). The study area covers a dimension of 113.39 km^2^ (City Population, 2023; Ghana Statistical Service (GSS), 2021). The research areas are considered economic hubs of the Greater Accra Region, with several industries and markets for suitable economic activities, coupled with residential facilities.

### Ethical considerations

The Environmental Health and Sanitation Directorate, Accra, Ghana, granted ethical approval for this study, reference AMA/OKS/EHSD/PH/26/07/01/2023. Participants signed written informed consent before responding to the questionnaire. Each respondent was assured of the confidentiality and anonymity of their personal information.

### Sample size determination

A random sampling method was used to select the target participant. The targeted sample size for DCB waste was 271, calculated using Cochran’s formula (Cochran, 1997):

$$n = {Z}^{2}pq/{e}^{2}$$

where *n* is the required sample size; *Z*^2^ is the 1.645 for a confidence level of 90%; *p* is the estimated proportion of the population (the presumed effect caused by DCB waste is assumed to be 50%, with *q* being *p* − 1); and *e* is the margin of error, set at 5%.

### Study design and data collection

A descriptive design approach was used for this study, and the research questionnaire was distributed using a simple random sampling method. Random sampling avoids or reduces selection bias (McEwan, 2020) and ensures data reliability and statistical representation (Uhunamure et al., 2021).

The questionnaire for the study was pre-tested in the Ashaiman community in Accra, Ghana, for seven days. It received 17 responses in the first week of December 2022, whose results were used to assess the clarity of questions and removed all identified ambiguity. The study was conducted from January to March 2023, with four hundred and twenty-one (421) participants voluntarily and freely participating in the research questionnaire. The inclusion criteria for the participants were as follows: (i) being 15 years and above, (ii) speaking English or any of the significant 45 Ghanaian local dialects (Dakubu, 1988), (iii) living in the study catchment area, and (iv) being mentally sound (one with right mind). similar to Mamady (2016). The exclusion criteria for the study included (i) individuals younger than 15 years, (ii) respondents residing outside the catchment area, and (iii) individuals with cognitive disabilities. The age 15 inclusion criteria were used for this study because individuals are in mid-adolescence, a period of learning behaviours leading to physical, emotional, and cognitive development. Hence, including ages 15 and above in the research captures a crucial transitional period in individuals’ lives and helps to gain insights that can inform future DCBWM strategies, education programmes, and community engagement efforts. Three hundred and sixty-seven (367) respondents were obtained from the participants, representing an 87.2% response rate, affirming the data's reliability and validity (Ellis et al., 2022). The study questionnaire focussed on the respondents’ socioeconomic status and demographic characteristics, that is, gender, educational level, family size, area of residents, income, and about knowledge of handling DCB waste from the point of generation to disposal, and effects on the environment and human health. The collected data were analysed using IBM Statistical Package for the Social Sciences (SPSS) version 27. The descriptive statistical methods such as frequency, mean, and percentage of the socio-demographic variables were calculated, with the influential factors of DCB waste analysed using a multivariate logistic regression model.

## Results

### Demographic characterization and socio-economic status of participants

Descriptive analysis in Table 1 shows that 367 participants responded to the survey questions, comprising 62.7% (230) males and 37.3% (137) females in a ratio of 1.78:1. The mean age of the respondents is 53.47, with the majority (47.1%) having primary education, 15.3% tertiary education and 5.7% no formal education. More than 70% reside in a planned residential area. The monthly income of the respondents ranges from USD 87 to USD 870 with a mean income of USD 270. Table 1 also identifies waste bins, burial, open burning, and open land space as a disposing source of DCB waste. 78% of the respondents disposed of DCB waste into waste bins, while 2% of the participants through open burning. The burning, burying, and open land space disposal methods of the DCB waste are done by respondents with some formal education who are primarily between the ages of 21 to 55 years and reside in a planned area.

**Table 1:** Socio-demographic characterization and dry cell battery waste disposal (*n* = 367)

Variable	*f* (%)	Dry cell batteries waste disposal method (*n* (%))			
		Waste bin	Buried	Open burning	Open land
Gender					
Male	230 (62.7)	185 (80.8)	8 (3.5)	2 (0.9)	20 (8.7)
Female	137 (37.3)	102 (74.5)	10 (7.3)	5 (3.6)	10 (7.3)
Education level					
None	21 (5.7)	5 (83.3)	–	–	–
Primary	173 (47.1)	15 (65.2)	–	–	6 (26.1)
Secondary	94 (25.6)	62 (72.1)	5 (5.8)	3 (3.5)	7 (8.1)
Tertiary	56 (15.3)	199 (81.6)	12 (4.9)	4 (1.6)	17 (7.0)
Tech. & voc	23 (6.3)	6 (85.7)	1 (14.3)	–	–
Age group					
< 20	6 (1.6)	18 (85.7)	3 (14.3)	–	–
21–35	23 (6.3)	133 (77.3)	7 (4.1)	4 (2.3)	13 (7.6)
36–45	86 (23.4)	80 (85.1)	3 (3.2)	1 (1.1)	5 (5.3)
46–55	245 (66.8)	39 (69.6)	3 (5.4)	2 (3.6)	9 (16.1)
> 55	7 (1.9)	17 (73.9)	2 (8.7)	–	3 (13.0)
Family size					
< 3	96 (26.2)	75 (78.1)	5 (5.2)	2 (2.1)	8 (8.3)
3–7	222 (60.5)	174 (78.7)	9 (4.1)	4 (1.8)	20 (9.0)
> 7	49 (13.4)	38 (77.6)	4 (8.2)	1 (2.0)	2 (4.1)
Residential area					
Unplanned	108 (29.4)	74 (68.5)	5 (4.6)	4 (3.7)	16 (14.8)
Planned	259 (70.6)	213 (82.6)	13 (5.0)	3 (1.2)	14 (5.4)
Monthly income (USD)					
< 87.1	116 (31.6)	87 (75.0)	8 (6.9)	1 (0.9)	10 (8.6)
87.1–217.5	107 (29.2)	82 (76.6)	3 (2.8)	1 (0.9)	12 (11.2)
217.6–348.0	57 (15.5)	43 (75.4)	5 (8.8)	3 (5.3)	4 (7.0)
348.1–522.0	30 (8.2)	22 (75.9)	1 (3.4)	2 (6.9)	2 (6.9)
522.1–696.0	23 (6.3)	21 (91.3)	–	–	1 (4.3)
696.1–870.0	17 (4.6)	16 (94.1)	1 (5.9)	–	–
> 870.0	17 (4.6)	16 (94.1)	–	–	1 (5.9)

*f* relative frequency, *n* sample dimension, *%* percentage, *USD* united State Dollar, *Tech & voc*. technical and vocational

### Use of dry cell batteries and segregation of its waste at home

The logistics regression model in Table 2 indicates that educational level, female gender, and residential area are likely to contribute to the use of DCB at home. However, educational variables such as secondary and tertiary education are the most significant predictors with odd ratios (OR) of 18.02 and 16.39, respectively. Also, the OR for female gender, educational level, age (under 20 and above 55), and monthly income indicate the probability of the participants segregating DCB waste at home.

**Table 2:** Socio-demographic factors influencing the use of dry cell batteries and its waste segregation at home

Variable	*f* (%)	Use dry cell batteries at home		Waste segregation	
		OR (95% CI)	*p*-value	OR (95% CI)	*p*-value
Gender					
Male	230 (62.7)	*Reference*		*Reference*	
Female	137 (37.3)	1.36 (0.73–2.54)	0.334	1.03 (0.58–1.84)	0.909
Age group					
< 20	21 (5.7)	*Reference*		*Reference*	
21–35	173 (47.1)	0.53 (0.04–6.86)	0.624	2.01 (0.50–8.02)	0.324
36–45	94 (25.6)	0.53 (0.58–4.78)	0.571	0.62 (0.19–1.97)	0.420
46–55	56 (15.3)	0.94 (0.10–8.84)	0.960	0.47 (0.17–1.54)	0.214
> 55	23 (6.3)	0.60 (0.06–5.97)	0.666	1.05 (0.33–3.34)	0.934
Education					
None	6 (1.6)	*Reference*		*Reference*	
Primary	23 (6.3)	2.59 (0.13–52.73)	0.537	2.01 (0.12–35.06)	0.633
Secondary	86 (23.4)	18.02 (1.104–294.27)	0.042*	1.38 (0.12–16.45)	0.797
Tertiary	245 (66.8)	16.39 (1.977–135.92)	0.010*	1.66 (0.168–16.39)	0.666
Tech. & voc	7 (1.9)	2.62 (0.41–16.71)	0.309	1.90 (0.204–17.72)	0.572
Residential area					
Unplanned residential area	108 (29.4)	*Reference*		*Reference*	
Planned residential area	259 (70.6)	1.25 (0.65–2.38)	0.507	0.71 (0.38–1.32)	0.274
Monthly income (USD)					
< 87.1	116 (31.6)	*Reference*		*Reference*	
87.1–217.5	107 (29.2)	0.22 (0.02–1.95)	0.173	1.12 (0.31–4.04)	0.868
217.6–348.0	57 (15.5)	0.36 (0.04–3.22)	0.361	0.58 (0.16–2.17)	0.418
348.1–522.0	30 (8.2)	0.46 (0.05–4.22)	0.490	1.56 (0.43–5.64)	0.500
522.1–696.0	23 (6.3)	0.49 (0.04–5.50)	0.563	1.26 (0.30–5.32)	0.750
696.1–870.0	17 (4.6)	1.30 (0.07–23.69)	0.859	1.43 (0.33–6.11)	0.633
> 870.0	17 (4.6)	0.95 (0.05–17.98)	0.973	1.16 (0.22–6.02)	0.858

OR > 1 implies that the event is more likely to happen; OR < 1 implies event is less likely to happen

*f* relative frequency, *OR* odds ratio, *95% CI* 95% confidence interval, *Tech. & voc.* technical and vocational, *%* percentage, *USD* United State Dollar

^*^p-value < 0.05

### Willingness to segregate dry cell battery waste and poor handling of its waste

From Table 3, the logistics regression model shows that the wiliness to segregate DCB waste likely depends on the female gender and income levels of the participants (OR > 1), with female gender being the most influential factor. However, female gender, age group, and educational status with the OR > 1 are likely to contribute to knowledge of handling DCB waste negatively affecting the environment. Participants with technical and vocational education have significant understanding of handling DCB waste.

**Table 3:** Factors influencing willingness to segregate and handling of dry cell batteries waste

Variables	*f* (%)	Willingness to segregate waste		Poor waste handling	
		OR (95% CI)	*p*-value	OR (95% CI)	*p*-value
Gender					
Male	230 (62.7)	*Reference*		*Reference*	
Female	137 (37.3)	1.79 (1.03–3.11)	0.040*	1.83 (0.86–3.92)	0.118
Age group					
< 20	21 (5.7)	Reference		Reference	
21–35	173 (47.1)	0.19 (0.017–2.21)	0.187	1.69 (0.21–13.61)	0.624
36–45	94 (25.6)	0.13 (0.015–1.16)	0.068	1.59 (0.35–7.25)	0.549
46–55	56 (15.3)	0.15 (0.016–1.31)	0.085	1.72 (0.38–7.71)	0.481
> 55	23 (6.3)	0.46 (0.049–4.37)	0.500	1.54 (0.32–7.39)	0.587
Educational level					
None	6 (1.6)	*Reference*		*Reference*	
Primary	23 (6.3)	0.000	0.999	2.52 (0.12–51.50)	0.548
Secondary	86 (23.4)	0.000	0.999	4.17 (0.408–42.63)	0.229
Tertiary	245 (66.8)	0.000	0.999	3.72 (0.517–26.75)	0.192
Tech. & voc	7 (1.9)	0.000	0.999	7.29 (1.08–49.20)	0.041*
Residential area					
Unplanned	108 (29.4)	*Reference*		*Reference*	
Planned	259 (70.6)	0.71 (0.40–1.26)	0.240	0.61 (0.27–1.34)	0.217
Monthly income (USD)					
< 87.1	116 (31.6)	*Reference*		*Reference*	
87.1–217.5	107 (29.2)	3.138 (0.79–12.53)	0.105	0.000	0.998
217.6–348.0	57 (15.5)	2.13 (0.55–8.21)	0.273	0.000	0.998
348.1–522.0	30 (8.2)	2.15 (0.525–8.84)	0.286	0.000	0.998
522.1–696.0	23 (6.3)	5.74 (0.99–32.99)	0.050	0.000	0.998
696.1–870.0	17 (4.6)	2.59 (0.45–14.91)	0.287	0.000	0.998
> 870.0	17 (4.6)	857,153,445.7 (0–0)	0.998	0.000	0.998

OR > 1 implies that the event is more likely to happen; OR < 1 implies event is less likely to happen

*f* relative frequency, *OR* odds ratio, *95% CI* 95% confidence interval, *Tech. & voc.* technical and vocational, *%* percentage, *USD* United State Dollar

^*^p-value < 0.05

### Indiscriminate disposal of dry cell battery waste and legislation regulating it as electronic waste in Ghana

The logistic regression model results in Table 4 show that female gender, age group, and monthly income are likely to influence the indiscriminate disposal of DCB waste, causing diseases. In relation to the legislation regulating electronic waste, such as DCB, income level and area of residence are likely to contribute to the model (OR > 1), with the female gender being the most significant predictor.

**Table 4:** Binary logistic regression model of association between indiscriminate disposal and law enforcement and socio-demographic characterization of respondents

Variables	*f* (%)	Indiscriminate disposal causing diseases		Laws that regulate dry cell battery waste	
		OR (95% CI)	*p*-value	OR (95% CI)	*p*-value
Gender					
Male	230 (62.7)	*Reference*		*Reference*	
Female	137 (37.3)	1.17 (0.65–2.11)	0.610	2.77 (1.63–4.72)	0.000*
Age group					
< 20	21 (5.7)	*Reference*		*Reference*	
21–35	173 (47.1)	2.26 (0.40–12.68)	0.354	0.83 (0.22–3.19)	0.787
36–45	94 (25.6)	0.86 (0.23–3.17)	0.820	0.51 (0.178–1.47)	0.215
46–55	56 (15.3)	1.22 (0.32–4.62)	0.774	0.67 (0.23–1.92)	0.458
> 55	23 (6.3)	1.44 (0.37–5.69)	0.600	1.04 (0.36–3.04)	0.939
Educational level					
None	6 (1.6)	*Reference*		*Reference*	
Primary	23 (6.3)	0.36 (0.03–4.31)	0.423	0.97 (0.09–11.89)	0.978
Secondary	86 (23.4)	0.58 (0.08–4.31)	0.594	1.49 (0.21–10.81)	0.690
Tertiary	245 (66.8)	1.77 (0.28–11.12)	0.544	0.84 (0.14–5.14)	0.849
Tech. & voc	7 (1.9)	2.34 (0.39–13.93)	0.349	1.43 (0.248–8.23)	0.689
Residential area					
Unplanned	108 (29.4)	*Reference*		*Reference*	
Planned	259 (70.6)	0.88 (0.47–1.63)	0.682	1.11 (0.65–1.88)	0.704
Monthly income (USD)					
< 87.1	116 (31.6)	*Reference*		*Reference*	
87.1–217.5	107 (29.2)	1.89 (0.50–7.09)	0.347	1.44 (0.44–4.68)	0.545
217.6–348.0	57 (15.5)	1.71 (0.46–6.31)	0.422	1.06 (0.33–3.41)	0.919
348.1–522.0	30 (8.2)	3.90 (0.9–16.83)	0.068	1.24 (0.38–4.07)	0.723
522.1–696.0	23 (6.3)	3.64 (0.72–18.49)	0.119	1.53 (0.42–5.60)	0.520
696.1–870.0	17 (4.6)	3.71 (0.58–23.71)	0.165	1.21 (0.32–4.63)	0.779
> 870.0	17 (4.6)	2.13 (0.37–12.21)	0.396	1.28 (0.29–5.65)	0.748

OR > 1 implies that the event is more likely to happen; OR < 1 implies event is less likely to happen

*f* relative frequency, *OR* odds ratio, *95% CI* 95% confidence interval, *Tech. & voc.* technical and vocational, *%* percentage, *USD* United State Dollar

^*^p-value < 0.05

### Recycling or reuse of dry cell battery waste

Table 5 represents the logistic regression analysis of indiscriminate disposal of DCB waste and its effects on the environment and the reuse or recycling of the waste. The OR of female gender, age group, family size, educational level, and monthly income are likely to change the effect of the handling and reuse of DCB.

**Table 5:** Association between the effect of indiscriminate disposal of dry cell and reuse/recycle batteries and socio-demographic conditions of participants

Variables	*f* (%)	Indiscriminate disposal dry cell harm environment		Reuse/recycling of dry cell battery waste	
		OR (95% CI)	*p*-value	OR (95% CI)	*p*-value
Gender					
Male	230 (62.7)	*Reference*		*Reference*	
Female	137 (37.3)	1.14(0.60–2.14)	0.695	1.49 (0.74–3.03)	0.267
Age group					
< 20	21 (5.7)	*Reference*		*Reference*	
21–35	173 (47.1)	0.89 (0.18–4.48)	0.893	0.20 (0.03–1.37)	0.102
36–45	94 (25.6)	1.08 (0.29–3.96)	0.907	0.43 (0.12–1.57)	0.200
46–55	56 (15.3)	1.00 (0.28–3.62)	0.996	0.35 (0.09–1.32)	0.121
> 55	23 (6.3)	1.21 (0.32–4.56)	0.777	0.346 (0.09–1.39)	0.136
Educational level					
None	6 (1.6)	*Reference*		*Reference*	
Primary	23 (6.3)	0.39 (0.02–6.43)	0.507	1.592 (0–0)	1.000
Secondary	86 (23.4)	0.57 (0.05–6.55)	0.648	130,087,576.3 (0–0)	0.999
Tertiary	245 (66.8)	0.879 (0.09–8.57)	0.912	529,018,594.3 (0 0)	0.999
Tech. & voc	7 (1.9)	0.864 (0.09–7.85)	0.897	241,310,281.4 (0 0)	0.999
Family size					
< 3	96 (26.2)	*Reference*		*Reference*	
3–7	222 (60.5)	2.32 (0.77–6.96)	0.134	1.66 (0.50–5.51)	0.406
> 7	49 (13.4)	1.09 (0.47–2.58)	0.828	1.43 (0.49–4.18)	0.507
Residential area					
Unplanned	108 (29.4)	*Reference*		*Reference*	
Planned	259 (70.6)	0.73 (0.37–1.43)	0.361	0.629 (0.288–1.374)	0.244
Monthly income (USD)					
< 87.1	116 (31.6)	*Reference*		*Reference*	
87.1–217.5	107 (29.2)	1.32 (0.29–6.02)	0.717	3.06 (0.34 –27.59)	0.320
217.6–348.0	57 (15.5)	1.56 (0.34–7.20)	0.567	1.73 (0.19–15.55)	0.623
348.1–522.0	30 (8.2)	1.11 (0.24–5.14)	0.893	3.09 (0.34–28.16)	0.316
522.1–696.0	23 (6.3)	0.91 (0.18–4.64)	0.908	2.44 (0.23–25.54)	0.460
696.1–870.0	17 (4.6)	0.71 (0.13–3.78)	0.691	8.64 (0.92–81.51)	0.060
> 870.0	17 (4.6)	1.08 (0.17–6.97)	0.933	5.75 (0.52–63.74)	0.154

OR > 1 implies that the event is more likely to happen; OR < 1 implies event is less likely to happen

*f* relative frequency, *OR* odds ratio, *95% CI* 95% confidence interval, *Tech. & voc.* technical and vocational, *%* percentage, *USD* United Statet Dollar

^*^p-value < 0.05

### Challenges associated with the management of dry cell battery waste

A critical examination of the study area reveals that the management of DCB waste presents a range of significant challenges. These challenges arise from a combination of infrastructural, financial, and social factors. One of the primary obstacles is the absence of adequate infrastructure to support the proper collection, segregation, and disposal of DCB waste. The lack of facilities and resources, common in the region, hinders efficient recycling and waste management efforts. Moreover, insufficient financial support for recycling programs exacerbates the problem, limiting the capacity of local authorities and organisations to implement sustainable waste management solutions. Without adequate funding, the necessary technology and logistics for handling DCB waste remain underdeveloped, leading to inefficiencies in the recycling process. Equally concerning is the lack of awareness within the community regarding proper DCB waste management practices. Public knowledge about the environmental and health risks associated with improper disposal of dry cell batteries is often limited, resulting in unsafe handling and disposal behaviours. The absence of widespread educational campaigns contributes to this gap in understanding, further complicating waste management efforts.

## Discussion

Disposing of DCB waste is a significant challenge in SSA, specifically in Ghana, as a result from the exponential procurement of electronic devices that use DCB (Liu et al., 2021; Karidis, 2021). The toxic content of the DCB waste negatively impacts the environment and threatens public health, i.e., SDGs 3, 6, 11, 12, 13, 14 and 15 (Debrah & Dinis, 2023; Karidis, 2021) due to improper disposal, example, burial, open land space, and use of waste bins. In light of this, a study was conducted to determine if socio-economic and demographic factors influence the knowledge of handling DCB waste in Accra, Ghana. The results of the study show that 70.6% of the respondents live in planned communities, with 78% of the participants disposing of DCB waste in the waste bin, while 8.2% dispose of them in the open land space, similar to the results obtained in a study conducted by Abdel-Shafy and Mansour (2018) and Ferronato and Torretta (2019) about the WM, also addressing DCB waste in developing countries. The improper disposal of the waste, including the DCB waste, will result in soil contamination (Ayilara et al., 2020; Cabrera, 2021), air and water pollution (Melchor-Martínez et al., 2021; Rajan et al., 2019), affecting environmental sustainability (Sharma et al., 2019; Siddiqua et al., 2022), contributing to non-achievement of individual good healthy well-being (SDG 3), clean water and sanitation (SDG 6), safe and sustainable cities for human settlement (SDG 11), sustainable consumption and production patterns (SDG 12), climate action (SDG 13), sustainable conservation of water (SDG 14) and better life on land (SDG 15) (Kopecká et al., 2024).

Generation of DCB waste could be influenced by socio-economic and demographic variables such as age, gender, monthly income, area of residence, and level of education in SSA (Lange et al., 2022). The multivariate linear regression analysis applied to the data of DCB waste shows that the demographic dynamics and socio-economic variables are likely to influence the management of this specific waste category. This study shows that gender (OR: 1.36), residential area (OR: 1.25), and monthly income levels (OR: 1.30) in the ranges of USD 696–USD 870 are likely to contribute to the use of DCB at home, with secondary and tertiary educational level being translated in an OR of 18.02 and 16.39, respectively, significantly influencing the use of a DCB, similar to studies carried out by Sawant et al. (2021), in India, and Trang et al. (2017), in Vietnam, on the effects of socio-economic factors on solid waste generation and composition. Also, 78.5% of the participants in this study do not practice DCB waste segregation. The small percentage of participants that practice segregation of DCB waste are females (OR: 1.03), within the age group of 21–35, and have some level of education, with better income, which agrees with the results from the study conducted by Babaei et al. (2015) on household recycling and segregation knowledge, attitudes and practices towards WM. According to Debrah et al. (2021b) and Arlinghaus and Johnston (2018), formal level of education creates awareness and behavioural change, enhancing waste segregation, including the case of DCB waste.

The results of this study show that gender and short monthly income are likely to contribute to the willingness to segregate DCB waste for disposal. However, the female gender and the high monthly income range of USD 522 and USD 696 significantly influence the willingness to segregate waste for disposal, results similar to the ones from Adzawla et al. (2019) research, reporting that high monthly income and female gender contribute to waste segregation, including DCB, for disposal. Nonetheless, the multivariate logistic regression model in this study seems to demonstrate that female gender is to be associated to the awareness of indiscriminate handling of waste. The study further indicates that the education level and age groups are likely to contribute to indiscriminate DCB waste disposal and its possible negative effects on human health, compromising SDGs 3, 6, 13, 14, and 15 (Leal Filho et al., 2022b, 2022e) by 2030. On the other hand, the indiscriminate disposal of DCB waste is less likely to be influenced by the level of education and area of residence, as shown in Table 5, similar to a study conducted by Mamady (2016), in Guinea, concerning factors influencing attitude, safety behaviour, and knowledge regarding household WM. To minimize indiscriminate DCB waste disposal, educational training on DCB waste awareness should be promoted (Amoah et al., 2023; Debrah et al., 2021b; Leal Filho et al., 2022c, 2022d), aiming for WM sustainability and a healthy environment. Also, the enforcement of legislation on DCB waste as electronic waste (Tanhaei et al., 2020) can help to regulate the disposal of DCB waste at the landfills (Moossa et al., 2023; Murthy & Ramakrishna, 2022).

In Africa, and as acknowledged by the scientific community, DCB waste recycling is a challenging issue due to inadequate infrastructure, absence of financial incentives and insufficient human resources (Debrah et al., 2022a, b, c, d, e; Dinis et al., 2022). The findings of this study show that 85% of the respondents do not recycle or reuse DCB waste, which corroborates the results of similar studies conducted by Adeleke et al. (2021), Almasi et al. (2019), Fakhri et al. (2021) and Karim and Corazzini (2019), where solid waste generated in households and the municipalities in most developing countries are neither recycled nor reused. According to Karidis (2021) and Rarotra et al. (2020) studies, recycling DCB waste is complex and cost-effective, requiring reliable infrastructure and recycling facilities. Recycling DCB contributes to preserve the natural environment and reduces pollution through recovering Hg from used DCB (Kang et al., 2023; Li et al., 2018) and recycling of outer Fe and residual Zn in the DCB into Fe products and Zn ingots (Kaya et al., 2020). Accordingly, many developing countries, especially in African regions, need clear action plans to manage and recycle electronic waste, including DCB (Asante et al., 2019), to reduce its indiscriminate disposal for a sustainable environment, aiming to advance the SDGs 3, 6, 11, 12, 13, 14 and 15. The multivariate logistics regression model carried out in this study suggests that the participants with high monthly income and high level of education are likely to recycle or reuse DCB, when there is provision of infrastructure such as recycling plants, which will convert the waste into useful material or purposes (Zhou & Wang, 2020). Demographic characteristics and socio-economic status are highly likely to correlate positively with the handling and management of DCB waste.

## Conclusions

The incorrect disposal of DCB waste is an issue in most developing countries, as the case of Ghana. Education and awareness on proper disposal of DCB waste and adequate regulation to protect the environment and human health need to be properly implemented. A descriptive and multivariate study was carried out within Accra-Tema metropolitan area in Ghana to ascertain the demographic characterisations and socio-economic dynamics that directly impact the management of DCB waste, with implications in terms of environmental sustainability. A random sampling technique and a descriptive cross-sectional survey were used to collect information from the 367 participants from January to March 2023. A significant number of the respondents were males, with a gender ratio (male to female) of 1.78:1, within the mean age of 53.47, with the majority residing in a planned area (70%). The results further revealed that most of the respondents disposed of DCB waste in waste bins (78%), with few either open land space (8%) or burying (5%) in a household. The multivariate logistics regression model implemented suggests that monthly income level and gender are influential factors in the wiliness to segregate DCB waste, with females being the significant predictor. Concerning the incorrect handling of DCB waste, it is clear that female gender, age group of 21 to 55, and higher educational level are parameters likely to positively influence the handling of this specific waste type. The results obtained demonstrate that participants lack the knowledge in properly handling DCB waste in Ghana households, resulting in indiscriminate disposal.

To the author’s best knowledge, the study focusing on demographic characterisations and socio-economic characteristics associated with the management of DCB waste in Ghana is the first of its kind. This study will contribute to assist in raising awareness of the proper management of DCB waste at home to reduce its indiscriminate disposal in Ghana, ensuring better health and a healthy environment. In addition, it is expected to encourage the Government of Ghana, and also other Governments of SSA Countries and relevant stakeholders, to the need to have access to the necessary infrastructure and technology for recycling and disposal of DCB waste, aiming to overcome the challenges associated with its improper handling in developing countries and advance the SDGs.

### Limitations and future prospects

One major setback of this study is that some of the participants did not respond to the open-ended questionnaires, probably resulting from busy work schedules, time constraints, or difficulty articulating their thoughts. This study must be conducted in entire regions of Ghana for further assessment of DCBWM, contributing to promoting change at the level of effective decision-making on WM.

## Data Availability

Data is available from the corresponding author upon reasonable request.

## Abbreviations

*DCB*: Dry cell battery
*WM*: Waste management
*OR*: Odd ratios
*SSA*: Sub-Saharan Africa
*SDGs*: Sustainable Development Goals
*SPSS*: Statistical Package for Social Sciences
*USD*: United States Dollar
